# Multiphase arterial spin labeling imaging to predict early recurrent ischemic lesion in acute ischemic stroke

**DOI:** 10.1038/s41598-022-05465-8

**Published:** 2022-01-27

**Authors:** Ki-Woong Nam, Chi Kyung Kim, Byung-Woo Yoon, Inpyeong Hwang, Chul-Ho Sohn

**Affiliations:** 1 Department of Neurology, Seoul Metropolitan Government-Seoul National University Boramae Medical Center, Seoul, Korea; 2 Department of Neurology, Seoul National University College of Medicine, Seoul, Korea; 3 Department of Neurology, Korea University Guro Hospital, 148 Gurodong-ro, Guro-gu, Seoul, 08308 Korea; 4 Department of Neurology, Korea University College of Medicine, Seoul, Korea; 5 Department of Neurology, Uijeongbu Eulji Medical Center, Eulji University College of Medicine, Uijeongbu-si, Korea; 6 Department of Radiology, Seoul National University Hospital, 101 Daehak-ro, Jongno-gu, Seoul, 03080 Korea; 7 Departement of Radiology, Seoul National University College of Medicine, Seoul, Korea; 8 Institute of Radiation Medicine, Seoul National University Medical Research Center, Seoul, Korea

**Keywords:** Neurology, Neurological disorders

## Abstract

In acute ischemic stroke (AIS), the hemodynamics around the lesion are important because they determine the recurrence or prognosis of the disease. This study evaluated the effects of perfusion deficits in multiphase arterial spin labeling (ASL) and related radiological parameters on the occurrence of early recurrent ischemic lesions (ERILs) in AIS. We assessed AIS patients who underwent multiphase ASL within 24 h of symptom onset and follow-up diffusion-weighted imaging within 7 days. ASL perfusion deficit, arterial transit artifact (ATA), and intra-arterial high-intensity signal (IAS) were manually rated as ASL parameters. A total of 134 patients were evaluated. In the multivariable analyses, ASL perfusion deficit [adjusted odds ratio (aOR) = 2.82, 95% confidence interval = 1.27–6.27] was positively associated with ERIL. Furthermore, when ATA was accompanied, the ASL perfusion deficit was not associated with ERIL occurrence. Meanwhile, IAS showed a synergistic effect with ASL perfusion deficit on the occurrence of ERIL. In conclusion, we demonstrated the association between perfusion deficits in multiphase ASL with ERIL in patients with AIS. This close association was attenuated by ATA and was enhanced by IAS. ASL parameters may help identify high-risk patients of ERIL occurrence during the acute period.

## Introduction

The recurrence of stroke increases the risk of disability and mortality, making it an important concern in patients with acute ischemic stroke (AIS)^1,2^. However, it is difficult to distinguish clinical recurrence through neurological examination in AIS patients with neurological deficits^1–3^. Although the reported clinical recurrence rate was < 5%^1,2^, early recurrent ischemic lesions (ERILs), defined as radiological recurrence on diffusion-weighted imaging, occurs up to 40% of patients within the first week after AIS^2,4^. Since the presence of symptoms in stroke is determined by the lesion size, location, and number, ERIL is considered a pathology such as clinical recurrence^4–6^. Therefore, as a surrogate of recurrent stroke, studies have identified various clinical, laboratory, and radiological predictors of ERIL^6–8^.

AIS is a vascular disease, in which the hemodynamics of the lesion area determine the disease prognosis or recurrence^9–12^. Therefore, it is important to accurately measure perfusion deficits or collateral flow in patients with AIS. Arterial spin labeling (ASL) is a noninvasive magnetic resonance imaging (MRI) technique that uses magnetically labeled blood as an endogenous contrast agent^13^. In previous studies, the perfusion deficit area on ASL was well correlated with the perfusion deficit area on conventional imaging techniques (e.g., MRI, computed tomography)^14–16^, and it was closely related to stroke prognosis and recurrence^13,17–20^.

In ASL imaging, not only the perfusion deficit but also the accompanying parameters are important^20^. When the time for labeled blood to reach the tissue (= arterial transit time, ATT) is longer than that of post-label delay (PLD) due to slow arterial flow, this delayed blood flow appears as a hyperintense signal on the ASL images^13,20,21^. These are called arterial transit artifact (ATA) (= cortical ATA) or intraarterial high-intensity signal (IAS) (= proximal ATA), depending on where they are found, indicating leptomeningeal collateral flow and stagnant flow by arterial occlusion, respectively^20–26^. ATA and IAS are frequently found in AIS patients, and their pathology has been clarified in several experimental studies^13,22–24,26^. However, studies on their effects on clinical prognosis are lacking.

Conventional ASL is a good technology, but, there are limitations in using it in the area where the ATT is normally long^22,27^. Multiphase ASL has addressed some of the issues with long-ATT areas by acquiring PLDs of multiple time points allowing the images both the early and late label arrival^13,22,27–30^. In this study, we attempted to demonstrate that ASL perfusion deficit has a positive correlation with ERIL occurrence in patients with AIS. Furthermore, we examined how other accompanying ASL parameters affect the association between perfusion deficits and ERIL. Since previous studies showed that the occurrence of ERIL differed according to the stroke mechanism^31^, we also compared the patterns of ASL parameters according to the stroke mechanism and the resulting ERIL occurrence.

## Results

A total of 134 AIS patients were assessed (mean age, 71 ± 13 years; male sex, 62.7%; mean initial NIHSS score, 6 ± 6). ERIL occurred in 59 (44.0%) patients, including 30 (22.4%) cases of ERIL-local and 29 (21.6%) cases of ERIL-distant. We identified ASL perfusion deficits in 87 (64.9%) patients, ATA in 37 (27.6%) patients, and IAS in 27 (20.1%) patients. Other detailed baseline characteristics are presented in Supplementary Table 1.

The baseline characteristics of the ERIL group did not differ from those of the no-ERIL group, except for higher frequencies of ASL perfusion deficits (74.6% versus 57.3%, *P* = 0.038) and IAS (33.9% versus 9.3%, *P* < 0.001, Table 1). In the multivariable analyses, ASL perfusion deficits (adjusted odds ratio [aOR] = 2.82, 95% confidence interval [CI] = 1.27–6.27) and age (aOR = 1.03, 95% CI = 1.00–1.06) were positively associated with ERIL after adjusting for confounders (Table 2).

**Table 1:** Baseline characteristics of patients with and without ERILs.

	No ERIL (n = 75)	ERIL (n = 59)	*P* value
Age, years [IQR]	71 [61–79]	74 [65–80]	0.218
Sex, male, n (%)	47 (62.7)	37 (62.7)	0.996
Follow-up MRI time, days [IQR]	2 [1–4]	3 [2–4]	0.520
Hypertension, n (%)	52 (69.3)	44 (74.6)	0.504
Diabetes, n (%)	29 (38.7)	24 (40.7)	0.813
Hyperlipidemia, n (%)	33 (44.0)	31 (52.5)	0.326
Atrial fibrillation, n (%)	23 (30.7)	19 (32.2)	0.849
Ischemic heart disease, n (%)	16 (21.3)	14 (23.7)	0.741
Smoking, n (%)	14 (18.7)	8 (13.6)	0.428
History of stroke, n (%)	19 (25.3)	14 (23.7)	0.831
**Mechanism, n (%)**			0.843
Intracranial-LAA	18 (24.0)	13 (22.0)	0.789
Extracranial-LAA	11 (14.7)	12 (20.3)	0.387
Cardioembolism	27 (36.0)	21 (35.6)	0.961
Cryptogenic	19 (25.3)	13 (22.0)	0.657
Initial NIHSS score [IQR]	4 [2–8]	3 [1–7]	0.091
IV thrombolysis, n (%)	11 (14.7)	5 (8.5)	0.272
HbA1c, % [IQR]	5.9 [5.5–6.5]	5.9 [5.7–6.9]	0.407
Fasting blood sugar, mg/dL [IQR]	98 [87–116]	98 [85–113]	0.857
Total cholesterol, mg/dL [IQR]	161 [142–182]	162 [137–202]	0.898
LDL cholesterol, mg/dL [SD]	97 ± 36	100 ± 37	0.659
HDL cholesterol, mg/dL [SD]	48 ± 14	46 ± 14	0.439
Triglyceride, mg/dL [IQR]	92 [67–124]	98 [76–153]	0.389
White blood cell, × 10^3^/μL [IQR]	7.15 [5.69–8.94]	6.97 [5.68–9.95]	0.761
High-sensitivity CRP, mg/dL [IQR]	0.11 [0.06–0.40]	0.11 [0.05–0.26]	0.655
ASL perfusion deficit, n (%)	43 (57.3)	44 (74.6)	0.038
ATA, n (%)	24 (32.0)	13 (22.0)	0.200
IAS, n (%)	7 (9.3)	20 (33.9)	< 0.001
Recanalization, n (%)	12 (16.0)	16 (27.1)	0.116

*ERIL* early recurrent ischemic lesion, *MRI* magnetic resonance imaging, *LAA* large artery atherosclerosis, *NIHSS* National Institutes of Health Stroke Scale, *IV*  intravenous, *HbA1c*  hemoglobin A1c, *LDL*  low-density lipoprotein, *HDL* high-density lipoprotein, *CRP*  c-reactive protein, *ASL*  arterial spin labeling, *ATA*  arterial transit artifact, *IAS*  intraarterial high-intensity signal.

**Table 2:** Multivariable logistic regression analysis of possible predictors for early recurrent ischemic lesion.

	Crude OR (95% CI)	*P*-value	Adjusted OR (95% CI)	*P*-value
Age, years	1.02 [1.00–1.05]	0.107	1.03 [1.00–1.06]	0.042
Sex, male	1.00 [0.50–2.03]	0.996	1.11 [0.53–2.32]	0.792
Initial NIHSS score	0.97 [0.92–1.03]	0.348	0.94 [0.88–1.01]	0.086
ASL perfusion deficit	2.18 [1.04–4.59]	0.040	2.82 [1.27–6.27]	0.011

*NIHSS* National Institutes of Health Stroke Scale, *ASL*  arterial spin labeling.

**Figure 1:**
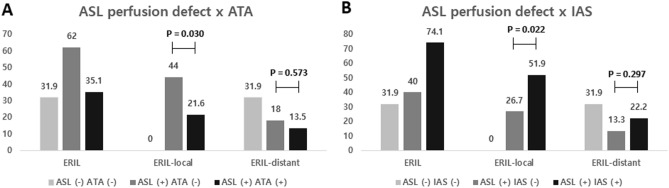
Effects of ATA and IAS on ERIL occurrence and locations. *ATA* arterial transit artifact, *IAS* intraarterial high-intensity signal, *ERIL* early recurrent ischemic lesion. (**A)** When accompanied by ATA, the incidence of ERIL-local was significantly lower in patients with ASL perfusion deficits (*P* = 0.030). However, in the case of ERIL-distant, the influence of ATA was unclear (*P* = 0.573). (**B)** Conversely, IAS significantly increased the incidence of ERIL-local in patients with ASL perfusion deficits (*P* = 0.022). Likewise, the impact of IAS on the occurrence of ERIL-distant was also less pronounced (*P* = 0.297).

Similar to previous studies, all ATA and IAS were found within the regions of the ASL perfusion deficit. Interestingly, additional multivariable analyses showed opposite effects on ERIL between ATA and IAS. For ATA, the [ASL deficit ( +) ATA (−)] group showed the highest frequency of ERIL (62.0%) and had the highest aOR value in multivariable analysis (aOR = 4.48, 95% CI = 1.83–10.98) (Table 3). Conversely, when accompanied by ATA, the ASL perfusion deficit lost its statistical significance with ERIL and showed an ERIL frequency similar to that in the [ASL deficit (−) ATA (−)] group (35.1% versus 31.9%, *P* = 0.756, Fig. 1). Thus, ATA appeared to negatively affect the occurrence of ERIL. Assessment of the effects of ATA according to the ERIL location showed, more ERIL-local in the [ASL deficit ( +) ATA (−)] group than that in the [ASL deficit ( +) ATA ( +)] group (44.0% versus 21.6%, *P* = 0.030). However, there was no significant difference in ERIL-distant between the two groups.

**Table 3:** Multivariable logistic regression analyses of possible predictors for ERIL, considering the interactive effects between ASL perfusion deficit and ATA/IAS.

	Crude OR (95% CI)	*P*-value	Adjusted OR (95% CI)	*P*-value
**Model 1**				
Age, years	1.02 [1.00–1.05]	0.107	1.03 [1.00–1.07]	0.039
Sex, male	1.00 [0.50–2.03]	0.996	1.07 [0.50–2.27]	0.869
Initial NIHSS score	0.97 [0.92–1.03]	0.348	0.95 [0.89–1.01]	0.108
**ASL deficit × ATA**		0.006		0.003
ASL deficit (−) ATA (−)	Ref	Ref	Ref	Ref
ASL deficit ( +) ATA (−)	3.48 [1.51–8.05]	0.004	4.48 [1.83–10.98]	0.001
ASL deficit ( +) ATA ( +)	1.16 [0.46–2.88]	0.756	1.47 [0.56–3.86]	0.436
**Model 2**				
Age, years	1.02 [1.00–1.05]	0.107	1.03 [1.00–1.07]	0.043
Sex, male	1.00 [0.50–2.03]	0.996	1.13 [0.52–2.42]	0.759
Initial NIHSS score	0.97 [0.92–1.03]	0.348	0.94 [0.88–1.01]	0.105
**ASL deficit × IAS**				0.001
ASL deficit (−) IAS (−)	Ref	Ref	Ref	Ref
ASL deficit ( +) IAS (−)	1.42 [0.64–3.17]	0.389	1.84 [0.78–4.31]	0.164
ASL deficit ( +) IAS ( +)	6.10 [2.12–17.54]	0.001	7.97 [2.59–24.51]	< 0.001

*ERIL*  early recurrent ischemic lesion, *ASL* arterial spin labeling, *ATA*  arterial transit artifact, *IAS*  intraarterial high-intensity signal, *NIHSS*  National Institutes of Health Stroke Scale.

In contrast, the [ASL deficit ( +) IAS ( +)] group showed the highest frequency of ERIL (74.1%) and the highest aOR value (aOR = 7.97, 95% CI = 2.59–24.51, Table 3). There were no significant differences between the [ASL deficit ( +) IAS (−)]and [ASL deficit (−) IAS (−)] groups (Fig. 1). These results indicated the positive effect of IAS on ERIL occurrence. Examination of the relationships between IAS and the ERIL location, showed more ERIL-local in the [ASL deficit ( +) IAS ( +)] group than in the [ASL deficit ( +) IAS (−)] group (51.9% versus 26.7%, *P* = 0.022). However, no significant differences were observed for ERIL-distant.

We compared the characteristics of the four groups of stroke mechanisms included in this study (Table 4). Although not statistically significant, ERIL most commonly occurred in the EC-LAA group (*P* = 0.843), while ERIL-local was more frequently found in the IC-LAA and EC-LAA groups (*P* = 0.029) and ERIL-distant showed a statistical tendency of increased frequency in CE or cryptogenic groups (*P* = 0.092). ASL perfusion deficits and ATA/IAS did not differ depending on the stroke mechanisms; however, recanalization was clearly observed in the CE group (*P* = 0.012).

**Table 4:** Comparisons of baseline characteristics, ASL parameters, and ERIL according to stroke mechanisms.

	IC-LAA (n = 31)	EC-LAA (n = 23)	CE (n = 48)	Cryptogenic (n = 32)	*P* value
ERIL	13 (41.9)	12 (52.2)	21 (43.8)	13 (40.6)	0.843
ERIL-local	10 (32.3)	9 (39.1)	6 (12.5)	5 (15.6)	0.029
ERIL-distant	3 (9.7)	3 (13.0)	15 (31.3)	8 (25.0)	0.092
ASL perfusion deficit	23 (74.2)	16 (69.6)	30 (62.5)	18 (56.2)	0.463
ATA	10 (32.3)	4 (17.4)	15 (31.2)	8 (25.0)	0.580
IAS	9 (29.0)	6 (26.1)	8 (16.7)	4 (12.5)	0.314
Recanalization	5 (16.1)	0 (0)	16 (33.3)	7 (21.9)	0.012
Age, years	76 [60–81]	75 [70–84]	76 [66–81]	70 [61–76]	0.025
Sex, male	16 (51.6)	17 (73.9)	31 (64.6)	20 (62.5)	0.401
Hypertension	21 (67.7)	21 (91.3)	32 (66.7)	22 (68.8)	0.149
Diabetes	15 (48.4)	9 (39.1)	17 (35.4)	12 (37.5)	0.702
Hyperlipidemia	15 (48.4)	16 (69.6)	17 (35.4)	16 (50.0)	0.061
Smoking	6 (19.4)	2 (8.7)	5 (10.4)	9 (28.1)	0.130
History of stroke	8 (25.8)	8 (34.8)	11 (22.9)	6 (18.8)	0.578
Initial NIHSS score	3 [2–6]	3 [1–4]	5 [2–11]	4 [1–6]	0.126
IV thrombolysis	2 (6.5)	1 (4.3)	8 (16.7)	5 (15.6)	0.310

*ASL*  arterial spin labeling, *ERIL*  early recurrent ischemic lesion, *IC-LAA* intracranial large artery atherosclerosis, *EC-LAA*  extracranial large artery atherosclerosis, *CE*  cardioembolism, *ATA*  arterial transit artifact, *IAS*  intraarterial high-intensity signal, *NIHSS*  National Institutes of Health Stroke Scale, *IV*  intravenous.

## Discussion

The results of this study showed the significant association of perfusion deficits on multiphase ASL with ERIL in patients with AIS. The close association was attenuated by ATA and enhanced by IAS. Thus, ASL perfusion deficits and accompanying radiological parameters may be helpful in identifying high-risk groups for ERIL occurrence in AIS patients.

Similar to our previous study on transient ischemic attack, we again confirmed the clinical significance of ATA and IAS^20^. Cortical ATA, which indicates rich leptomeningeal collateral flow, attenuates ischemic core progression and reduces the final infarct volume^19,21,29,32^. In our results, ATA showed a protective effect on the occurrence of ERIL. This effect was related only to ERIL-local occurring within the region where the collateral flow was distributed, but not to ERIL-distant. Meanwhile, IAS refers to stagnant flow due to large vessel occlusion^24,25^. Therefore, whether it is the actual recurrence due to embolism or in situ thrombosis, or a silent recurrence due to the breakup of the initial thrombi, it seems natural that IAS is positively correlated with ERIL.

ERIL occurred in different patterns according to the stroke mechanisms. Using the accompanying radiological parameters, we were able to speculate on the mechanism by which these differences occurred. Unsurprisingly, ERIL mainly appeared in the form of ERIL-local within the relevant vessel area in the IC-LAA and EC-LAA groups. There were three cases of exceptional ERIL-distant, in which there was no initial ASL perfusion deficit or when the embolism was transferred to the contralateral anterior cerebral artery territory along the circle of Willis (Supplementary Table 2). Thus, all ERILs occurring in IC-LAA and EC-LAA patients can be interpreted as recurrent local problems of relevant vessels. However, in the CE and cryptogenic groups, ERIL-local and ERIL-distant were mixed, suggesting that embolisms were the main mechanisms.

Detailed examination of the mechanism showed the highest frequency of ASL perfusion deficits in the IC-LAA; however, its effect on ERIL occurrence was not statistically significant (Supplementary Table 2). The frequency of ATA was also the highest, with only two of 10 patients with ATA showing ERIL, underscoring the protective effect. IC-LAA also had the highest frequency of IAS (Table 4). Thus, the incidence of ERIL in the IC-LAA group could be attributed to various mechanisms (e.g., artery-to-artery embolism, in situ thrombosis, and the breakup of initial thrombi)^33^. In the case of EC-LAA, all ERIL occurred only in relevant vessels that were advanced enough to accompany the ASL perfusion deficit (Supplementary Table 2). In addition, since there were no recanalization events in this group, all ERILs were thought to have occurred due to true recurrence events. Therefore, artery-to-artery embolism is likely the main mechanism of ERIL occurrence in EC-LAA as revealed in previous studies^33^. CE patients had a significantly higher recanalization rate (Table 4) and ERIL developed in 10 of these 16 patients with recanalization. In other words, there seemed to be a high rate of silent recurrence due to the breakup of the initial thrombi^31^. However, only four such cases in the present study occurred in the form of ERIL-local, while six were accompanied by ERIL-distant; therefore, the effect of additional embolic events cannot be ignored. Patients with cryptogenic stroke did not show distinct features, likely be due to the mixture of heterogeneous mechanisms (e.g., paradoxical embolism, paroxysmal atrial fibrillation, malignancy) in this patient group.

Despite the novel findings in this study, there are several limitations to consider when interpreting our results. First, this was a single-center, retrospective, cross-sectional study. Given the limitations of the cross-sectional analysis, our findings indicated associations rather than causal relationships. Second, ERIL used as an outcome variable did not consider the recurrence of clinical symptoms. Therefore, some of these instances may have been caused by the breakup of initial thrombi, regardless of actual recurrence events. However, in previous studies, ERIL greatly affected clinical recurrence and subsequent prognosis^2,4^. Third, in this study, ASL perfusion deficit, ATA, and IAS were measured manually by neuroradiologists. If these parameters were rated in an automatic way, the reproducibility would be higher. Fourth, we conducted this study using only qualitative ASL parameters. If we verify our study using quantitative parameters such as spatial coefficient of variation in future studies, higher reliability could be obtained^34^. Lastly, this study included only patients with ischemic stroke in the anterior circulation. With the advent of multiphase ASL technology, the resolution problem of ASL has significantly improved. However, due to the technical limitations of ASL images, their use in posterior circulation stroke remains limited.

The results of this study demonstrated that perfusion deficits on multiphase ASL was associated with ERIL in patients with AIS in the anterior circulation. In addition, using the accompanying ATA and IAS, we estimated the risk of ERIL and predicted the underlying pathophysiology of ERIL occurrence according to stroke mechanisms. These ASL parameters will help us to identify groups at high-risk for ERIL occurrence during the acute period and to perform early interventions according to the mechanisms. However, further prospective studies are required to confirm our findings.

## Methods

### Study population

We evaluated AIS patients who underwent multiphase ASL between April 2014 and May 2020. This study only included AIS of the anterior circulation (e.g., anterior/middle cerebral artery, internal carotid artery) (n = 331). This is considering the natures of the ASL image, in which the posterior circulation has relatively low resolution and it is difficult to measure ATA and IAS. Patients were then excluded according the following criteria: (1) visited 24 h after symptom onset (n = 7); (2) no follow-up MRI within 7 days (n = 81); and (3) administration of diagnostic or therapeutic intraarterial thrombolysis (n = 20)^4,31^. Similar to previous studies on ERIL, we also excluded patients with the following stroke mechanisms (n = 89): (1) small vessel occlusion, (2) other determined, and (3) more than two stroke mechanisms^31^. However, in recent years, the clinical importance of cryptogenic stroke has been highlighted; therefore, we included this group of patients, unlike previous studies. Thus, the final analysis included 134 patients with AIS.

This retrospective cross-sectional study was approved by the Institutional Review Board (IRB) of Seoul National University Hospital (IRB number: 1103-135-357), which waived the requirement for written informed consent due to the retrospective study design and the use of de-identified and anonymized patient information only. All experiments were conducted in accordance with the Declaration of Helsinki and all relevant guidelines and regulations. All data related to this study are included in the main text and supplemental materials.

### Clinical assessment

In our center, all AIS patients were principally admitted by their physicians for a broad evaluation to identify the stroke etiology and prognosis. The demographic, clinical, and cardiovascular risk factors, assessed in this study were age, sex, hypertension, diabetes, dyslipidemia, atrial fibrillation, ischemic heart disease, current smoking, history of stroke, initial stroke severity, mechanisms of stroke, and use of intravenous thrombolysis^20^. Initial stroke severity was assessed using the National Institutes of Health Stroke Scale (NIHSS) score evaluated every day from admission to discharge by well-trained neurologists who were not involved in this study. The mechanism of stroke was determined according to the Trial of ORG 10172 in Acute Stroke Treatment classification^35^. Based on this, we classified our study population into four groups as follows: intracranial large artery atherosclerosis (IC-LAA), extracranial large artery atherosclerosis (EC-LAA), cardioembolism (CE), and cryptogenic^31^. IC-LAA was defined as symptomatic intracranial atherosclerosis (occlusion or ≥ 50% stenosis) without evidence of EC-LAA or CE^31^. EC-LAA was diagnosed when patients had symptomatic extracranial atherosclerosis without IC-LAA or CE^31^. Laboratory examinations were performed after a 12 h overnight fast, included glucose profiles, lipid profiles, white blood cell counts, and high-sensitivity C-reactive protein^20^.

### Radiological assessment

**Figure 2:**
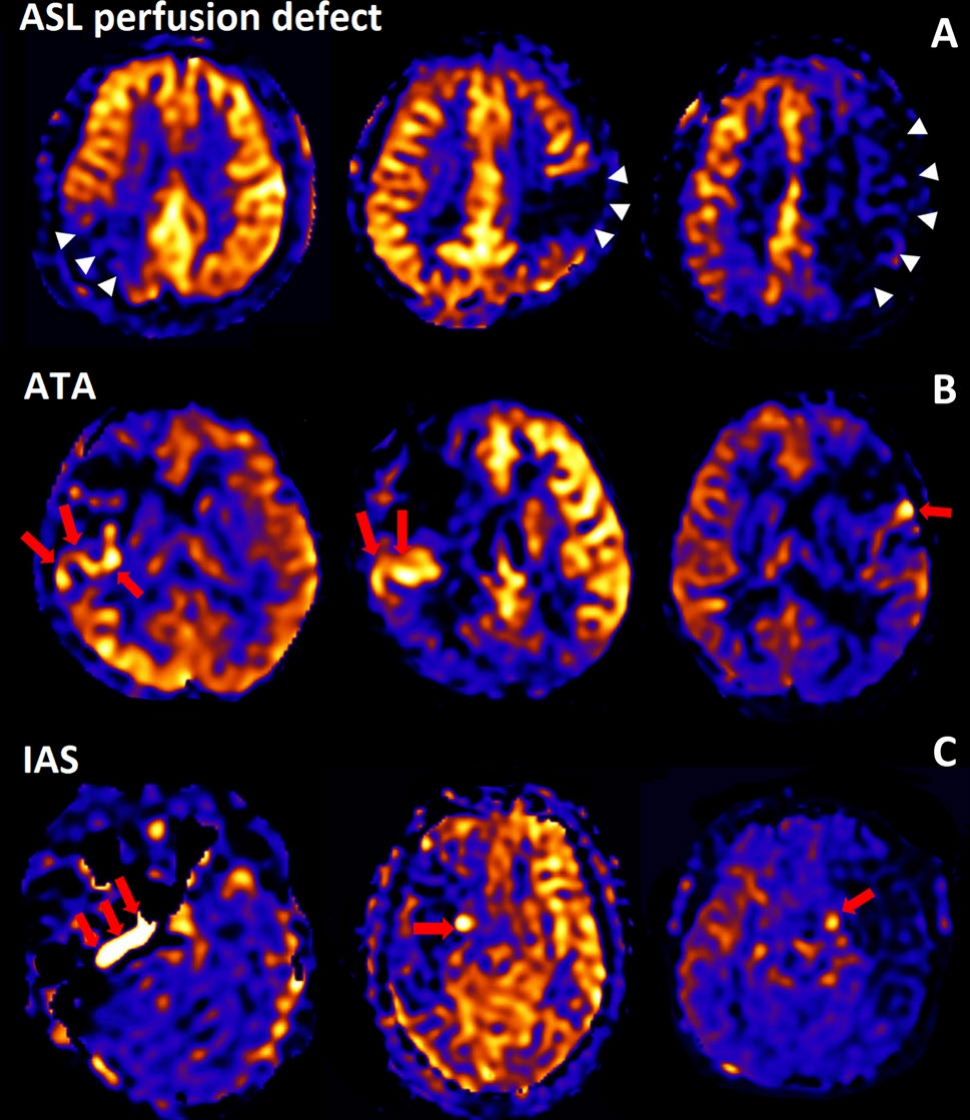
Representative cases of arterial spin labeling (ASL) perfusion deficit, arterial transit artifact (ATA), and intraarterial high-intensity signal (IAS) on multiphase ASL images. **(A)** ASL perfusion deficit, **(B)** arterial transit artifact, **(C)** intraarterial high-intensity signal.

All participants in this study underwent brain MRI and magnetic resonance angiography (MRA) within 24 h of admission and follow-up MRI within 7 days using a 3.0-T MR scanner (Discovery MR750w; GE healthcare, Milwaukee, WI, USA) with a 32-channel phased-array head coil. Basal MP-ASL perfusion-weighted MRI was obtained using three-dimensional (3D) spiral fast spin-echo sequences with a Hadamard-encoded pseudo-continuous ASL^36,37^. The imaging parameters were as follows: repetition time, 5902.0 ms; echo time, 11.3 ms; slice thickness, 5 mm; number of averages, 1; number of slices, 28; readout, 4 spiral arms × 640 samples; field of view, 24 × 24 cm^2^; matrix, 128 × 128; and voxel resolution, 3.8 mm × 3.8 mm × 5.0 mm. The protocol encoded 7 different PLD times into a single acquisition. With the parameters tabulated above, images with PLD times of [1.00, 1.22, 1.48, 1.78, 2.15, 2.63, and 3.32] seconds and effective label durations of [0.22, 0.26, 0.30, 0.37, 0.48, 0.68, and 1.18] seconds were reconstructed. These PLD times are intended to probe the bolus arrival time. The ATT map (δ) and perfusion map (f) were calculated by fitting the seven-delay ASL difference signals as a function of the PLD (w) to the following equation:

$$ \Delta {\text{M}}\, = \,{\text{2M}}^{0}_{{\text{t}}} \cdot \beta \cdot \alpha \cdot {\text{T1t}} \cdot {\text{f}} \cdot {\text{e}}^{{ - \delta /{\text{T1a}}}} \cdot \left( {{\text{e}}^{{ - {\text{max}}\left( {{\text{w}} - \delta ,0} \right)/{\text{T1t}}}} {-}{\text{e}}^{{ - {\text{max}}(\tau + {\text{w}} - \delta ,0)/{\text{T1t}}}} } \right)/\lambda^{{{23},{37},{37}}} $$

where *ΔM* is the ASL difference signal, *f* is the perfusion rate, *T1a* and *T1t* are the longitudinal relaxation times of blood and tissue, *M*^*0*^_*t*_ is the fully relaxed equilibrium magnetization of brain tissue, *α* is the efficiency of the labeling sequence, *λ* is the tissue-to-blood partition coefficient of water, *δ* is the transit delay, *τ* is the labeling duration, and *w* is the PLD. Vascular signal suppression is assumed in this model. *β* has been added to the kinetic model to compensate for any static tissue signal loss caused by the vessel suppression pulses^36,38–41^. From this map, an ASL perfusion deficit was defined based on qualitative visual interpretation by two well-trained neuroradiologists (IP.H. and C.-H.S.) who were blinded to the other clinical information (Fig. 2). Other radiological parameters related to ASL (ATA and IAS) were also measured as in our previous study (Fig. 2)^29^. ATA was defined as a focus or curvilinear hyperintensity located bordering the areas of ASL perfusion deficits^25^. IAS was a hyperintense signal appearing within the vascular lumen, directly proximal or distal to the relevant occluded vascular lesions^15^. Recanalization was rated based on the initial and follow-up MRA, including both partial and complete recanalization^31^.

As an outcome variable, ERIL was defined as a new diffusion-weighted imaging lesion outside the index symptomatic lesion area on initial MRI^1^. An enlargement of the index lesion was not considered an ERIL^1^. We also classified ERIL into two groups—ERIL-local and ERIL-distant—according to the relationship between the locations of the ERIL and the initial ASL perfusion deficit (Supplementary Fig. 1). ERIL was classified as ERIL-local if new lesions occurred within the initial ASL perfusion deficit area and as ERIL-distant if they occurred outside the area^1^. ERILs occurring simultaneously in and out of the ASL perfusion deficit were classified as ERIL-distant, as previously described^1^. All radiological parameters were visually analyzed by well-trained neuroradiologists (IP.H. and C.-H.S.), and disagreements were resolved by discussion with a third rater (C.K.K.).

### Statistical analysis

All statistical analyses were performed using IBM SPSS Statics for Windows, version 23.0 (IBM Corp., Armonk, NY, USA). Univariate analyses for evaluating the possible predictors for ERIL were performed using Student’s *t* or Mann–Whitney *U*-tests for continuous variables and chi-square or Fisher’s exact test for categorical variables. Variables with *P* < 0.10 in the univariate analyses, as well as age and sex, were included in the multivariable logistic regression analysis.

Based on their definitions, ATA and IAS are only found in patients with ASL perfusion deficits^20^. To interpret the individual or mutual meanings of these parameters, we classified the study population into three groups according to the presence of ASL perfusion deficit and ATA/IAS (e.g., [ASL deficit × ATA] or [ASL deficit × IAS]). Using these multi-categorical variables, we performed an additional multivariable logistic regression analysis. We also analyzed the relationships between these variables and ERIL-local/ERIL-distant to understand the effect of these radiological parameters on the location of the ERIL occurrence.

This study included patients with four different stroke mechanisms. Thus, we also compared baseline characteristics, ASL parameters, and ERIL among patient groups according to stroke mechanism. In this study, all variables with *P* < 0.05 were considered statistically significant.

## Supplementary Information

Supplementary Information.
