# Supplementary material for: Multiphase arterial spin labeling imaging to predict early recurrent ischemic lesion in acute ischemic stroke
Source: Sci Rep. 2022 Jan 27;12:1456. doi: 10.1038/s41598-022-05465-8 (PMC8795409; doi:10.1038/s41598-022-05465-8)
Supplement: Supplementary file 1 — Supplementary Information. [file 41598_2022_5465_MOESM1_ESM.pdf]

Supplemental Materials

Supplementary Figure 1. Representative cases of ERIL-local and ERIL-distant

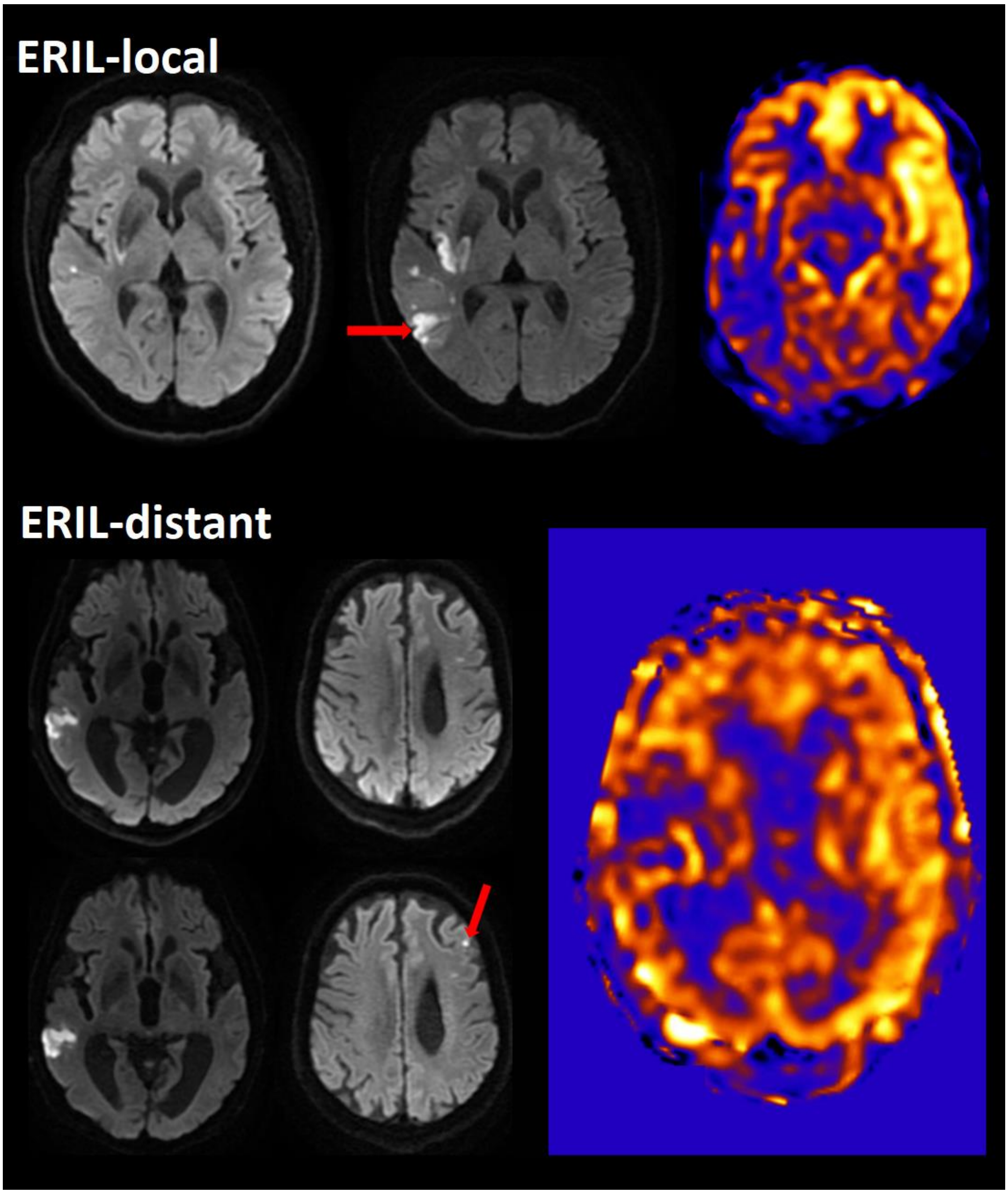

ERIL-local) On follow-up MRI, a new lesion appears only in the right M2 inferior division area, the area where the initial ASL perfusion deficit was located, for which it was classified as ERIL-local.

ERIL-distant) Follow-up MRI shows a new lesion in the left MCA area, in which no initial ASL perfusion deficit was noted initially. We classified this patient as ERIL-distant.

Supplementary Table 1. Baseline characteristics of the study cohort (n = 134)

**Demographic and clinical factors**	
Age, y [SD]	71 ± 13
Sex, male, n (%)	84 (62.7)
Follow-up MRI time, d [SD]	3 ± 2
Hypertension, n (%)	96 (71.6)
Diabetes, n (%)	53 (39.6)
Hyperlipidemia, n (%)	64 (47.8_
Atrial fibrillation, n (%)	42 (31.3)
Ischemic heart disease, n (%)	30 (22.4)
Smoking, n (%)	22 (16.4)
History of stroke, n (%)	33 (24.6)
Mechanism, n (%)	
Intracranial-LAA	31 (23.1)
Extracranial-LAA	23 (17.2)
Cardioembolism	48 (35.8)
Cryptogenic	32 (23.9)
Initial NIHSS score [SD]	6 ± 6
IV thrombolysis, n (%)	16 (11.9)
**Laboratory factors**	
HbA1c, % [SD]	6.4 ± 1.2
Fasting blood sugar, mg/dL [SD]	105 ± 34
Total cholesterol, mg/dL [SD]	167 ± 41
LDL cholesterol, mg/dL [SD]	98 ± 36
HDL cholesterol, mg/dL [SD]	47 ± 14
Triglyceride, mg/dL [SD]	113 ± 65
White blood cell, x 10^3/μL [SD]	7.65 ± 2.60
High-sensitivity CRP, mg/dL [SD]	0.45 ± 1.40
**Radiological factors**	
ERIL, n (%)	59 (44.0)
ERIL-local, n (%)	30 (22.4)
ERIL-distant, n (%)	29 (21.6)
ASL perfusion deficit, n (%)	87 (64.9)
ATA, n (%)	37 (27.6)
IAS, n (%)	27 (20.1)

Recanalization, n (%)	28 (20.9)

MRI = magnetic resonance imaging, LAA = large artery atherosclerosis, NIHSS = National Institutes of Health Stroke Scale, IV = intravenous, LDL = low-density lipoprotein, HDL = high-density lipoprotein, CRP = C-reactive protein, ERIL = early recurrent ischemic lesion, ASL = arterial spin labeling, ATA = arterial transit artifact, IAS = intraarterial high-intensity signal

Supplementary Table 2. Effects of ASL parameters on the occurrence of early recurrent ischemic lesion

	IC-LAA	EC-LAA	CE
ASL perfusion deficit	0	↑↑↑	0
Arterial transit artifact	↓	0	0
Intraarterial high-intensity signal	↑	↑	↑
Recanalization	0	**No case**	↑
Suspected mechanism	In situ thrombosis<br>Artery-to-artery embolism<br>Break-up of initial thrombi	Artery-to-artery embolism	Cardioembolism<br>Break-up of initial thrombi

ASL = arterial spin labeling, IC-LAA = intracranial large artery atherosclerosis, EC-LAA = extracranial large artery atherosclerosis, CE = cardioembolism

↑ Positive correlation, $P < 0.10$; ↑↑↑ Positive correlation, $P < 0.05$

↓ Negative correlation, $P < 0.10$; **0** Not statistically significant
